# Do general intellectual functioning and socioeconomic status account for performance on the Children's Gambling Task?

**DOI:** 10.3389/fnins.2013.00068

**Published:** 2013-06-03

**Authors:** Fernanda Mata, Isabela Sallum, Débora M. Miranda, Antoine Bechara, Leandro F. Malloy-Diniz

**Affiliations:** ^1^Laboratório de Investigações Neuropsicológicas, Faculdade de Medicina, Instituto Nacional de Ciência e Tecnologia de Medicina Molecular, Universidade Federal de Minas GeraisBelo Horizonte, Brazil; ^2^Faculdade de Medicina, Instituto Nacional de Ciência e Tecnologia de Medicina Molecular, Universidade Federal de Minas GeraisBelo Horizonte, Brazil; ^3^Department of Psychology, Brain and Creativity Institute, University of Southern CaliforniaLos Angeles, CA, USA

**Keywords:** intelligence, SES, affective decision-making, preschoolers and cognitive development

## Abstract

Studies that use the Iowa Gambling Task (IGT) and its age-appropriate versions as indices of affective decision-making during childhood and adolescence have demonstrated significant individual differences in scores. Our study investigated the association between general intellectual functioning and socioeconomic status (SES) and its effect on the development of affective decision-making in preschoolers by using a computerized version of the Children's Gambling Task (CGT). We administered the CGT and the Columbia Mental Maturity Scale (CMMS) to 137 Brazilian children between the ages of 3 and 5 years old to assess their general intellectual functioning. We also used the Brazilian Criterion of Economic Classification (CCEB) to assess their SES. Age differences between 3- and 4-years-old, but not between 4- and 5-years-old, confirmed the results obtained by Kerr and Zelazo (2004), indicating the rapid development of affective decision-making during the preschool period. Both 4- and 5-years-old performed significantly above chance on blocks 3, 4, and 5 of the CGT, whereas 3-years-old mean scores did not differ from chance. We found that general intellectual functioning was not related to affective decision-making. On the other hand, our findings showed that children with high SES performed better on the last block of the CGT in comparison to children with low SES, which indicates that children from the former group seem more likely to use the information about the gain/loss aspects of the decks to efficiently choose cards from the advantageous deck throughout the task.

## Introduction

Cognitive ability and achievement throughout life has been intensely linked with socioeconomic status (SES) (Bradley and Corwyn, 2002; Noble et al., 2005). For instance, SES has a significant impact on early reading ability, even when controlling for phonological awareness (Noble et al., 2006). In terms of achievement, the risk for children from low SES background to face school problems, such as grade repetition and dropping out of school is twice as high as in children from high SES background, and almost 50% higher for having a learning disability (Duncan and Brooks-Gunn, 2000). Furthermore, in a study carried out by Lawlor et al. (2006), family income in the year of the birth and parental education—indices related to SES—and breast feeding were the strongest predictors of intelligence at age 14. The persistence of poverty across generations may be a result of this gap in cognitive achievement between children from low and high SES (Farah et al., 2006). According to Bradley and Corwyn (2002), low SES represents an important disadvantage in childhood since it is associated with negative effects not only on cognitive attainment but also with socio-emotional development. They stated that there is evidence that children from low SES more frequently present symptoms of psychopathology and maladaptive social functioning than children from high SES background although the association between SES and children's social and emotional development is not as consistent as the relationship with cognitive and academic attainment.

The influence of SES across the different neurocognitive systems is inconclusive and controversial. For example, Noble et al. (2007) propose that SES has a distinct impact over different executive functions (EF), defined as the cognitive functions that underlie goal-directed behavior (Barkley, 1997). Noble et al. (2007) found that SES was related to both the working memory and the cognitive control system and those relations were fully mediated by either home and school environment (regarding working memory) or language skills (regarding cognitive control). However, the reward processing system was not found to be associated with SES or any environmental variable, suggesting a complex interplay between SES and neurocognitive systems. In another study, Noble et al. (2005) reported that 5-years-old from low and middle SES were equally inclined to delay rewards during a delay of gratification task. According to Farah et al. (2006), SES has a different impact over the underlying neural circuits of different neurocognitive systems depending on the specificity of each neural circuitry. In those terms, they argue that the circuitry underlying reward processing (orbitofrontal cortex—OFC) develops earlier than the other circuits of the prefrontal cortex, and thus SES would have a smaller impact over emotional cognition. Furthermore, while some studies have found SES factors to be significantly associated with performance on complex decision-making tasks in pre-adolescents (Gao et al., 2012) and with a delay of gratification task in adolescents (Anokhin et al., 2011) and adults (Green et al., 1996), other studies failed to find such association in kindergarteners (Noble et al., 2005), school-aged children (Noble et al., 2007), and adolescents (Olson et al., 2007).

To our knowledge, no study has assessed the relation between SES and affective decision-making in kindergartens (Zelazo and Muller, 2002; Kerr and Zelazo, 2004). Affective decision-making are decisions with emotional consequences that are marked by meaningful rewards and/or losses (Kerr and Zelazo, 2004). The process of decision making is also argued to demand more purely cognitive skills, such as attention and working memory, necessary to keep track over the consequences of previous choices (Dretsch and Tipples, 2008). Making decisions that will bring greater long-term gains instead of immediate rewards is a crucial skill that develops throughout childhood (Garon and Moore, 2004) and adolescence (Prencipe et al., 2011), and it can predict future adaptive behaviors (Mischel et al., 1988; Eigsti et al., 2006; McCabe et al., 2006; Casey et al., 2011).

The Iowa Gambling Task (IGT) is the most widely used paradigm to assess whether adolescents and adults are able to use past experiences to make advantageous decisions in the long run (Buelow and Suhr, 2009). Low performance on this task was first exhibited by patients with ventromedial prefrontal cortex (VM-PFC) lesions who exhibited real-world decision-making deficits but who did well on other tests of EF (Bechara et al., 1994). Nonetheless, recent studies have found that working memory and attention are also important for performance on the IGT (Hongwanishkul et al., 2005; Dretsch and Tipples, 2008). Furthermore, Yechiam et al. (2005) argued that performance on the IGT is related to attention to gains and losses, attention to recent outcomes and response sensitivity. Therefore, performance on the IGT encompasses both emotional and cognitive processes.

Many studies have investigated the development of decision-making in kindergarten children, school-aged children and adolescents and have suggested a progressive development of the ability to make advantageous choices over the course of the task, resulting in greater long-term gains (Mata et al., 2011). Some studies that used a child-friendly version of the IGT to assess affective decision-making in young children demonstrated that there are marked improvements in affective decision-making during the preschool period (Kerr and Zelazo, 2004; Bunch et al., 2007; Gao et al., 2009). For example, Kerr and Zelazo (2004) used the Children's Gambling Task (CGT), a two-deck version of the IGT, in which the number of happy faces on each card indicated the number of rewards (M&M chocolate candies) gained, whereas the number of sad faces indicated the number of rewards lost. Although 3-years-old children selected more cards from the disadvantageous deck, 4-years-old children chose more cards from the advantageous deck when compared to chance. The results of the study by Gao et al. (2009) confirmed those findings. It should be noted that findings from studies using the IGT and its variants as indices of affective decision-making during childhood and adolescence are marked by significant individual differences in scores (Evans et al., 2004; Kerr and Zelazo, 2004; Balodis et al., 2006; Huizenga et al., 2007; Hooper et al., 2008). For example, although Kerr and Zelazo (2004) found evidence of a significant improvement in performance on the CGT between the ages of 3 and 4, the authors noticed considerable variability within each age group. Gao et al. (2009) also noted important individual differences in performance on this gambling task. This performance variability for the preschool years may be a result of several factors, such as SES and general intellectual level.

In this sense, there have been countless debates on the extent to which general intellectual functioning may be related to performance on affective decision-making tasks. Although many studies have failed to find any significant correlations between intellectual performance on tasks which are used to assess affective decision making (Crone and van der Molen, 2004; Hooper et al., 2004, 2008; Hongwanishkul et al., 2005; Brand et al., 2007; Toplak et al., 2010), other studies have found an association between intellectual levels and performance on these tasks (Demaree et al., 2010; Schutter et al., 2010; Suhr and Hammers, 2010; Willner et al., 2010). Some studies support the notion that performance on the affective decision-making processes in childhood and adolescence and general intellectual functioning seem to be relatively dissociated (Crone and van der Molen, 2004; Hooper et al., 2004; Hongwanishkul et al., 2005; Brand et al., 2007; Toplak et al., 2010).

To our knowledge, Hongwanishkul et al. (2005) was the only study that investigated the association between intellectual ability and performance of preschoolers on an affective decision-making task. They found that performance on the CGT and the Delay of Gratification Task were unrelated to general intellectual functioning in 3- to 5-years-old children. In that study, however, they used the Peabody Picture Vocabulary Test, a measure of receptive vocabulary that evaluates primarily crystallized intelligence, to evaluate intellectual functioning. The cognitive processes that may be related to performance on gambling tasks are likely to be the ones associated with fluid intelligence; therefore, we must further investigate such interactions in preschool children.

It is important to note that almost all of the studies concerning the relationship between SES and EF were conducted in developed countries (e.g., Noble et al., 2005; Farah et al., 2006; Biro et al., 2009). Noble et al. (2007) suggest that it is important to investigate if these results could be generalized to other cultures in which there is more heterogeneity in the distribution of SES across the population. The present study aimed to investigate whether SES and general intellectual functioning were associated with affective decision-making in young children. In this way, we aimed to explain the significant individual differences in scores that characterize preschoolers' performance on gambling tasks. We hypothesized that performance on the task used to assess affective decision-making would be related to general intellectual functioning and SES. Furthermore, since general intellectual functioning is argued to be associated with SES (Duncan et al., 1994), it was important to have an intellectual functioning measure to control its impact on performance on the affective decision-making task.

## Methods

### Participants

We tested 137 healthy children between the ages of 3 and 5 years old. There were 37 children aged 3 years (mean age = 44.05 months, *SD* = 2.581, 19 males, 23 children with high SES), 50 children aged 4 years (mean age = 53.64 months, *SD* = 3.515, 29 males, 26 with high SES) and 50 children aged 5 years (mean age = 65.24 months, *SD* = 3.36, 29 males, 26 with high SES) from three public and three private kindergartens in Belo Horizonte, Brazil. All participants were normally developing children and were from different socioeconomic backgrounds, as shown in Table 1.

**Table 1:** **Socioeconomic characteristics of children from high and low SES^a^**.

	**Children from public kindergartens (*N* = 59)**	**Children from private kindergartens (*N* = 75)**	***t or χ^2^***	***p* (two-tailed)**
	**Mean (*SD*) or Count (Percentage)**	**Mean (*SD*) or Count (Percentage)**		
Score – Number of resources at home	6.85	13.09	13.56	0.000
**YEARS OF SCHOOLING OF THE HOUSEHOLDER**				
0–3	1 (1.8)	1 (1.3)	66.71	0.000
4	11 (19.6)	0		
8	24 (42.9)	4 (5.3)		
12	17 (30.4)	19 (25.3)		
16 or more	3 (5.4)	51 (68.0)		
**NUMBER OF PEOPLE LIVING IN THE CHILD'S HOUSE**				
0–4	35 (64.8)	33 (82.7)	10.87	0.15
5 or more	19 (35.3)	13 (17.2)		
Total score on the CCEB	17.31	32.48	16.15	0.000

a Measured by the Brazilian Criterion of Economic Classification (CCEB).

### Measures

#### The Brazilian Criterion of Economic Classification (CCEB)

SES was assessed using the Brazilian Criterion of Economic Classification (CCEB), according to the criteria established by the Brazilian Research Enterprises Association (ABEP, 2008). In Brazil, the CCEB is a widely used questionnaire in social and behavioral research that estimates the purchase power of families living in urban areas. It includes nine items that assess the available resources at home and one item that assesses the education level of the householder, resulting in a scale ranging from 0 to 46 points. Families are categorized into eight economic classes, from top to bottom: A1 (42–46 points), A2 (35–41), B1 (29–34), B2 (23–28), C1 (18–22), C2 (14–17), D (8–16), and E (0–7). These classes are further divided into two main economic classes: high (A and B) and low (C, D, and E). The average monthly income for families classified as A (A1 and A2) is U$4337.14 and B (B1 and B2) is U$1599.84. The average monthly income for families classified as C (C1 and C2), D, and E are U$632.5, U$351.72, and U$234.97, respectively. The CCEB also assesses the number of people living in the child's house and identifies the homeowner of the family. Data for six children (three 4-years-old children from private kindergartens: two girls and one boy, and three children from public kindergartens: two 3-years-old girls and one 4-years-old girls) were missing because their parents did not fill out the questionnaire.

#### Children's Gambling Task-Br (CGT-Br)

**Figure 1:**
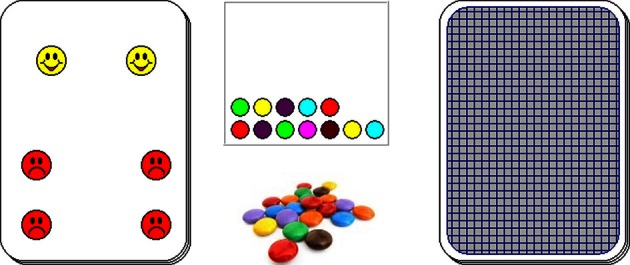
**Sample card from the disadvantageous deck of the Children Gambling Task-Br**.

Affective decision-making was measured by the CGT—Brazilian Version (CGT-Br). The difference between the CGT (Kerr and Zelazo, 2004) and its Brazilian version lies in their manual and computerized applications, respectively. The CGT-Br (Figure 1) includes two decks of fifty-three cards each and a box placed between them representing the 10 ml glass cylinder that holds the candies, which is presented on the center of a white computer screen. The back of one deck is covered with black diagonal lines, while the back of the other deck is covered with horizontal and vertical lines. The front of the cards from both decks is then divided in half. Before the experimenter reveals the bottom half of each card, he needs to click on the post-it to reveal any sad faces, which simulates the study conducted by Kerr and Zelazo (2004). Cards from the deck with the diagonal lines always provide two rewards, which are represented by happy yellow faces, and either zero or one loss, which are represented by sad red faces. Cards from the deck with horizontal and vertical lines always provide two rewards and losses of 0, 4, 5, or 6 candies. Therefore, the deck with diagonal lines is advantageous in the long-run, whereas the deck with horizontal and vertical lines is disadvantageous. The order of the cards in each deck was the same as that of Kerr and Zelazo (2004), as shown in Table 2.

**Table 2:** **Outcomes associated with each card in the advantageous and disadvantageous decks**.

**Card n°**	**1**	**2**	**3**	**4**	**5**	**6**	**7**	**8**	**9**	**10**	**11**	**12**	**13**	**14**	**15**	**16**	**17**	**18**	**19**	**20**	**21**	**22**	**23**	**24**	**25**
Disadvantageous deck losses	0	0	−4	0	−6	0	−4	0	−5	−6	0	−6	0	−5	−4	0	−6	−4	0	0	0	−6	0	−6	0
Advantageous deck losses	0	0	−1	0	−1	0	−1	0	−1	−1	0	−1	−1	0	0	0	−1	−1	0	−1	0	0	0	−1	−1
**Card n°**	**26**	**27**	**28**	**29**	**30**	**31**	**32**	**33**	**34**	**35**	**36**	**37**	**38**	**39**	**40**	**41**	**42**	**43**	**44**	**45**	**46**	**47**	**48**	**49**	**50**
Disadvantageous deck losses	−4	−5	−4	0	0	−6	−4	−5	0	0	0	−4	−6	0	0	0	0	−4	0	−6	0	−4	0	−5	−6
Advantageous deck losses	−1	0	0	−1	−1	0	0	0	−1	−1	0	−1	0	−1	−1	0	0	−1	0	−1	0	−1	0	−1	−1

Procedures and instructions were based on Kerr and Zelazo (2004). The rewards were M&Ms, the same used in the original study. After giving one reward to motivate children to play, the demonstration trials were started. In this stage, the experimenter would make three consecutive choices from the disadvantageous deck, follow by three consecutive choices from the advantageous deck, showing the children the gain and losses for each choice. Following the demonstration trials, an initial stake of 10 M&Ms was given to the children. The task would then be started and the children had to choose across 50 test trials, which were divided into five blocks of ten card choices. In both demonstration and test trials, the experimenter announced the number of candies won, placed them on the happy faces and then put them on the table next to the computer. In addition, candies were added to a glass cylinder presented on the computer screen to force children to focus on the candies gained. When children lost rewards, candies were removed, placed on the top of the sad faces and returned to a yellow box. The removal of candies could also be observed in the cylinder on the computer screen. At the end of the game, the children were asked which deck was better and why, and they received a packet of M&Ms candies for their participation independent of the amount of M&Ms they gathered in the task. It is worthy of note that participants were instructed that they would win the amount of candies they gathered as Kerr and Zelazo (2004) did, but that all participants won the same amount of M&Ms. This information was clarified in the informant consent sent to the parents. As the dependent variable, we used the proportion of advantageous choices minus the number of disadvantageous choices made throughout the 50 trials.

#### Columbia Mental Maturity Scale (CMMS)

The general intellectual ability of the participants was measured using the Columbia Mental Maturity Scale (CMMS) (Burgemeister et al., 1954). The CMMS is a measure of non-verbal cognition, which is related to different measures of intelligence, such as the Raven's Progressive Matrices and the Wechsler Intelligence Scale for Children (Barratt, 1956). The CMMS is designed to evaluate children between the ages of 3 years and 6 months to 9 years and 11 months old. It includes 92 items organized into eight age levels (A, B, C, D, E, F, G, and H); the child performs the segment of the test according to his or her chronological age. Therefore, depending on the level of the child, 55 to 66 items are presented. Each item includes a series of three to five concrete or abstract pictures printed on a card of 6 × 9 in. For each item, the child must point to the picture that is not related to the other ones. Because the number of items is different depending on the level of the child, scores were converted to *z-scores* relative to the distribution of the children, considering their ages by 6 to 6 months. Nine children (three 3-years-old girls, two 3-years-old boys, two 4-years-old girls, one 4-years-old boy, and one 5-years-old girl) from public schools were excluded because they could not understand the examples. These children were not part of the 137 children who participated.

## Procedures

An informed consent form was sent to the parents of the children with the help of school's teachers and coordinators. After the parents filled out the form, the children were tested using the CGT-Br and the CMMS. The tests were applied individually in a 50-min session in a quiet room of their school. Half of the children were first evaluated by the CGT-Br, whereas the other half began with the CMMS. The children were allowed to have a short break before beginning the second task. In addition, parents were also required to fill out the CCEB, which was sent together with the informed consent form.

This study was approved by the Research Ethics Committee of the Federal University of Minas Gerais (ETIC 511-09-UFMG), Belo Horizonte, MG, Brazil.

## Results

The data were analyzed as proposed by Kerr and Zelazo (2004). The primary dependent measure was whether children made an advantageous or disadvantageous choice in each trial. The 50 test trials were divided into five blocks of 10 cards each. Proportion scores were used to analyses performance across blocks. The proportion of advantageous choices per block minus the proportion of disadvantageous choices per block was computed for each of the five blocks in the task. Positive differences in scores revealed more advantageous choices, whereas negative differences revealed more disadvantageous choices. Means and standard deviation by SES groups are shown in Table 3.

**Table 3:** **Descriptive statistic for the CGT-Br net score by SES and age groups**.

	**Population**			**High SES**			**Low SES**		
	**3 years-old (*n* = 37)**	**4 years-old (*n* = 50)**	**5 years-old (*n* = 50)**	**3 years-old (*n* = 23)**	**4 years-old (*n* = 26)**	**5 years-old (*n* = 26)**	**3 years-old (*n* = 14)**	**4 years-old (*n* = 24)**	**5 years-old (*n* = 24)**
Mean	−1.30	5.12	6.36	0.35	7.31	5.46	−4.00	2.86	7.33
*SD*	7.50	7.68	9.16	6.46	9.21	10.35	8.52	5.04	7.77

Differences in scores were analyzed using a 3 (age: 3 vs. 4 vs. 5 years) × 5 (blocks 1–5) × 2 (high SES vs. low SES) mixed model analysis of variance (ANOVA). Since the performance on CMMS was found to be lower for the low SES group in comparison to the high SES group, *t*_(132)_ = −4.186, *p* < 0.001, we used performance on the CMMS as a covariate on the mixed model analysis. This analysis showed a significant effect of age *F*_(2, 105)_ = 10.55, *p* < 0.001 and block *F*_(4, 420)_ = 6.19, *p* < 0.001, which indicates that performance on the CGT-Br is associated with age, and that the number of advantageous minus disadvantageous choices differs among the blocks of the task independently of the other variables. The main effect of SES barely missed reaching the conventional significance level, *F*_(1, 105)_ = 13.8, *p* = 0.07. This analysis also showed a significant Age × Block interaction, *F*_(3.074, 322.77)_ = 4.63, *p* < 0.001 and a significant Block × SES interaction *F*_(3.074, 322.77)_ = 3.84, *p* = 0.01 (Greenhouse-Geisser adjusted). No other significant interactions were found and there was no main effect of CMMS over the model. Sex was not included in the model because its effect on performance on the CGT-Br and its interaction with performance on the CMMS and SES were insignificant.

**Figure 2:**
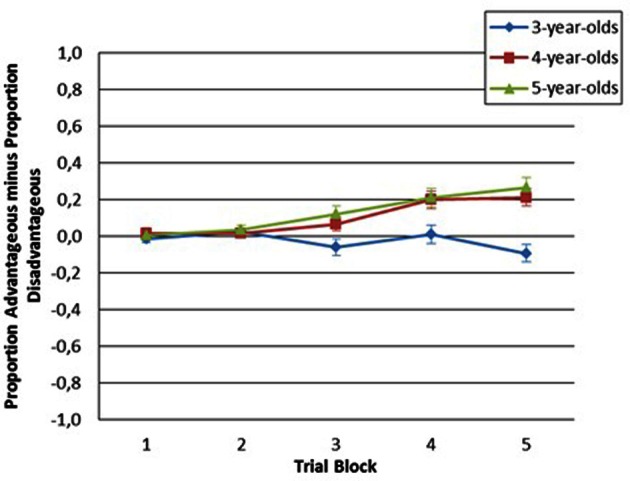
**Performance of each of the 3 aged groups across blocks**.

**Figure 3:**
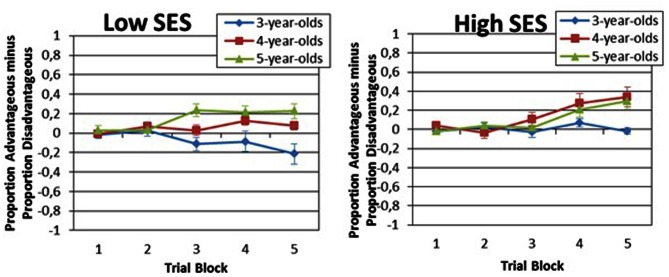
**Performance of each of the 3 aged groups according to SES groups**.

Tests of simple effects indicated that scores increased across blocks for 4-years-old, *F*_(3.074, 150.626)_ = 8.64, *p* < 0.001 and 5-years-old, *F*_(3.074, 115.275)_ = 8.31, *p* < 0.001, but not for 3-years-old, *F*_(3.074, 110.664)_ = 1.97, *p* = 0.113 (Greehouse-Geisser adjusted). Children aged 5 made more advantageous choices than did children aged 3 on block 5, *F*_(1, 85)_ = 20.88. It also revealed statistical trends for the blocks 3, *F*_(1, 85)_ = 7.75, *p* = 0.007, and 4, *F*_(1, 85)_ = 7.66, *p* = 0.007. To correct for the effect of multiple tests on the probability of a type I error, a significance cut-off of *p* < 0.003 was adopted, which was related to the Bonferroni correction for five tests (0.05/15 = 0.003). The same significance value was adopted for comparing block performances between 3- and 4-years-old children. Children aged 4 made more advantageous choices than did children aged 3 on block 5, *F*_(1, 85)_ = 20.46, *p* < 0.001. It also revealed statistical trend for the block 4, *F*_(1, 85)_ = 9.13, *p* = 0.003. Conversely, 4- and 5-years-old children performed similarly on all blocks of the task. Mean values and standard errors for each of the 5 blocks for each age group are presented in Figures 2, 3.

We used the *t*-distribution to compare mean values for each age group and blocks to the expected mean values based on random responding (i.e., 0). Three-years-old mean scores did not differ from chance. Four-years-old mean scores were significantly higher than chance for blocks 4, *t*_(49)_ = 4.18, *p* < 0.001, and 5, *t*_(49)_ = 4.55, *p* < 0.001. Five-years-old mean scores were significantly higher than chance for blocks 3, *t*_(49)_ = 2.69, *p* = 0.01, 4, *t*_(49)_ = 4.16, *p* < 0.001, and 5, *t*_(49)_ = 4.59, *p* < 0.001.

Because the Block × SES interaction *F*_(3.074, 322.77)_ = 3.84, *p* = 0.01 (Greenhouse-Geisser adjusted) was significant, we proceeded to make a series of comparisons between low and high SES children. Children from the high SES group made more advantageous choices than children from the low SES group only on the last block of the task, *F*_(1.537, 80.692)_ = 5.17, *p* = 0.025. Mean values and standard errors for each of the 5 blocks for each age group and SES are presented in Figure 3.

To investigate children's awareness of the game, they were asked the following question by the experimenter after they finished the task: “Which deck is the best to pick cards from?” Among 3-years-old, 16 of 35 children selected the advantageous deck (13 high SES children, 10 boys). Among four-year-olds, 25 of 47 children selected the advantageous deck (15 high SES children, 14 boys). Among 5-years-old, 26 of 50 children selected the advantageous deck (14 high SES children, 16 boys). A chi-square analysis of the children who chose the advantageous deck as the answer showed that the percentage of high SES children was higher than the percentage of low SES children χ^2^(1, *N* = 67) = 4.31, *p* = 0.038.

We performed an age-partialled correlation to test whether performance on CMMS was related with the CGT net score. This correlation failed to reach significance (*r* = 0.075, *p* = 0.387).

## Discussion

The present study is, to our knowledge, the first to investigate the association between socioeconomic levels and the development of affective decision-making during the preschool years. A computerized version of the CGT (Kerr and Zelazo, 2004) was developed for this purpose. Overall performance did not differ statistically significantly between SES groups. This is consistent with the notion of an early maturation of the VM-PFC (Fuster, 2002), which is responsible for CGT performance, and is consequently less influenced by SES factors. However, our findings showed that children with high SES performed better on the last block of the gambling task in comparison to children with low SES. This finding may be interpreted as the high SES children's stronger ability to inhibit and recall previous experiences of gains/losses and to adjust their performance across trials on the basis of information about past results. According to Gao et al. (2009), the ability to learn the gain/loss contingency is important when predicting future scenarios and being motivated by the affective aspects of those representations. Furthermore, the better performance of the high SES group in the last block of the task may also be related to other components of EF whose development may contribute to performance on the affective decision-making task, such as working memory (Hongwanishkul et al., 2005). It is according to an additional relationship between decision-making task performance and later developing control processes depending on the maturation of the dorsolateral prefrontal cortex (DL-PFC).

As mentioned previously, few studies have analyzed the association between SES and the affective/motivational aspects of EF. Anokhin et al. (2011) used the delay discounting paradigm in a sample of twin adolescents from different SES and found that adolescents from lower-income families tended to choose more immediate, but smaller rewards, in comparison to adolescents from higher-income families. The authors proposed a causal relationship between the tendency to discount delayed rewards and SES, arguing that such a relationship is genetically mediated because individuals who are genetically predisposed to making impulsive decisions are more likely to be from lower-income families. Nonetheless, the way the environment and biology interact, along with cognitive development, is still unclear, and epigenetic mechanisms can also mediate such relationships.

A strong association between performance on EF tasks and socioeconomic background is expected (Noble et al., 2005, 2007) due a greater susceptibility to environmental influences presented by the brain systems with protracted postnatal development such as the prefrontal regions that underlie executive functioning (Casey et al., 2000). However, a number of studies failed to find a relationship between SES and delay of gratification tasks, whereas a relationship was found between the more purely cognitive aspects of EF such as working memory and SES factors (Noble et al., 2005, 2007; Farah et al., 2006). According to Farah et al. (2006), a neurobiological hypothesis that could explain this dissociation is that throughout brain development, the VM-PFC, which is related to the more motivational/affective EF, is characterized by earlier maturation in comparison to the DL-PFC, which is related to the purely cognitive EF, such as working memory, inhibitory control, mental flexibility which are less related to emotional and motivational processes (Fuster, 2002). Therefore, it is possible that a longer period of development leaves the DL-PFC more susceptible to the numerous environmental influences that accompany SES. The current finding that CGT performance is associated to SES raises several questions that need to be addressed in future studies. For instance, the relationship between the development of the different sectors of the prefrontal cortex and the possible influence of the environment still needs to be investigated.

The differences between our results and those reported by Farah et al. (2006) might be explained by the assessment method used. Farah et al. (2006) used the delay of gratification task, which is simpler than the CGT. The CGT involves cost-benefits analysis and decision under ambiguity and risk. Furthermore, it is important to note that some studies have reported that affective decision making, as assessed by IGT and its variants for evaluation in childhood and adolescence, is in some way associated with working memory (Bechara et al., 2000; Hongwanishkul et al., 2005) and inhibitory control (Crone et al., 2005), and the DL-PFC would thus have a greater impact over this task.

Another possible explanation for our results might be related to the association between SES and differences in declarative memory development (Noble et al., 2005, 2007), a cognitive function subserved by the hippocampus and important for creating and adapting the relational representation between the decks and its rewards and punishments in the IGT (Gupta et al., 2009). Gupta et al. (2009) found that participants with amnesia due to bilateral hippocampal damage were unable to choose more cards from the advantageous decks in the gambling task suggesting that the hippocampus also contributes to advantageous decision-making as measured by the IGT. It is thus possible that the differences in the declarative memory development between children from low and high SES can explain the better performance of the latter in the CGT-Br. Future studies should address this issue. It is also possible that the children who participated in our study were from a lower socioeconomic home and school environment in comparison to those of Farah et al. (2006).

In the present study, performance on the task improved significantly between 3- and 4-years-old but not between 4- and 5 years-old. Four-year-olds performed significantly above chance on blocks 4 and 5 whereas 5-years-old performed significantly above chance on blocks 3, 4, and 5 of the CGT-Br. Three-years-old mean scores did not differ from chance. Therefore, it is important to note that the difference between age groups seems to be mostly driven by the difference between children who favored the advantageous deck and those who chose randomly. Brazilian children between the ages of 4 and 5 years old completed the computerized task in the same way as children who were tested in previous studies that used the original version of the CGT (Kerr and Zelazo, 2004; Hongwanishkul et al., 2005). This finding is consistent with the notion that the affective components of EF develop rapidly during the first 5 years of life (Zelazo and Muller, 2002) and may reflect continuing growth of the neural systems involving the OFC (Kerr and Zelazo, 2004). Our findings may also be interpreted when considering the development of working memory, attention and inhibitory control in this period and their impact on performance on gambling tasks (Hongwanishkul et al., 2005).

The results of the present study demonstrated that performance on the CMMS, a measure of fluid intelligence used to evaluate general intellectual functioning, was not related to affective decision-making. This finding was similar to those found in previous studies (Crone and van der Molen, 2004; Hongwanishkul et al., 2005; Brand et al., 2007; Hooper et al., 2008; Toplak et al., 2010). An explanation for the lack of association between general intellectual functioning and the CGT-Br presented in our study can be found in Hongwanishkul et al. (2005). These authors argued that although the more purely cognitive aspects of EF may be related to standard measures of intelligence, the affective components of the EF such as affective decision-making may be associated with emotional intelligence. It is also important to note that the use of a fluid intelligence measure to analyze the association between general intellectual functioning and affective decision-making in the present investigation makes an addition to the previous study with preschoolers that examined such association using a crystallized intelligence measure (Hongwanishkul et al., 2005).

Although the present study suggests possible differences in performance on the CGT-Br for children from different socioeconomic backgrounds, these results should be interpreted with caution because we only considered the number of resources at home, years of schooling of the homeowner and the number of people living in the child's house as SES factors. Furthermore, as in previous studies, the present study found notable individual differences in performance on the CGT-Br within each age group. Therefore, in addition to general intellectual functioning and socioeconomic factors, the influence of other potential contributors to performance on gambling tasks, such as personality, working memory, inhibitory control, delayed gratification, other EF, understanding of probabilities and parental engagement of children in everyday decision-making (Xiao et al., 2011) should be studied to investigate the great individual variability that characterizes performance on the CGT. Investigation of such performance variability in preschoolers may have an important role in understanding the processes behind affective decision-making. Another limitation of our study is that we had a smaller three-years-old group size compared to the other age groups, which may indicate low statistical power. It did not appear to be a critical issue, however, because we found significant differences between the different age groups.

In conclusion, regardless of the limitations already mentioned, the present study made important contributions to our understanding of the development of affective decision-making in young children. Our study did not find a relationship between affective decision-making and general intellectual functioning. On the other hand, our results indicated that differences in performance on affective decision-making tasks are related to socioeconomic background. Such knowledge may provide a new, specific target for intervention programmes in early childhood. As suggested by Noble et al. (2005), many randomized and controlled trials have demonstrated that intervention is capable of reducing the differences in performance between children from different socioeconomic backgrounds. Furthermore, it is important to note that intervention programmes are also an experimental design that permits researchers to test hypotheses about SES and affective decision-making. By using efficient intervention programmes, future studies can determine whether SES is related to the more affective or merely cognitive components of affective decision-making. In addition to genetic factors, future studies should also seek more specific causal factors in the environments of children from low socioeconomic background, such as parental stress, availability and parenting techniques that could be responsible for the results that we found.

### Conflict of interest statement

The authors declare that the research was conducted in the absence of any commercial or financial relationships that could be construed as a potential conflict of interest.
